# Trehangelins ameliorate inflammation-induced skin senescence by suppressing the epidermal YAP-CCN1 axis

**DOI:** 10.1038/s41598-022-04924-6

**Published:** 2022-01-19

**Authors:** Mami Yokota, Yoshiyuki Kamiya, Tamie Suzuki, Shinsuke Ishikawa, Akira Takeda, Shinya Kondo, Takeshi Tohgasaki, Takuji Nakashima, Yoko Takahashi, Satoshi Ōmura, Tetsuhito Sakurai

**Affiliations:** 1 FANCL Research Institute, FANCL Corporation, 12-13 Kamishinano, Totsuka-ku, Yokohama, Kanagawa Japan; 2 Department of Plastic and Aesthetic Surgery, Kitasato University School of Medicine, 1-15-1 Kitasato, Minami-ku, Sagamihara, Kanagawa Japan; 3 Research Organization for Nano and Life Innovation, Waseda University, 530 Wasedatsurumaki-cho, Shinjuku-ku, Tokyo Japan; 4 Ōmura Satoshi Memorial Institute, Kitasato University, 5-9-1 Shirokane, Minato-ku, Tokyo Japan

**Keywords:** Skin diseases, Senescence

## Abstract

Trehangelins (THG) are newly identified trehalose compounds derived from broth cultures of an endophytic actinomycete, *Polymorphospora rubra*. THG are known to suppress Cellular Communication Network factor 1 (CCN1), which regulates collagen homeostasis in the dermis. Although the physical properties of THG suggest a high penetration of the stratum corneum, the effect of THG on the epidermis has not been reported. Here we describe a possible mechanism involved in skin aging focusing on the effect of THG on epidermal CCN1. This study shows that: (1) THG suppress epidermal CCN1 expression by inhibiting the translocation of Yes-Associated Protein (YAP) to nuclei. (2) Epidermal CCN1, localized at the basement membrane, regulates the balance between the growth and differentiation of keratinocytes. (3) Keratinocytes secrete more CCN1 than fibroblasts, which leads to disruption of the basement membrane and extracellular matrix components. (4) The secretion of CCN1 from keratinocytes is increased by ultraviolet B exposure, especially in aged keratinocytes, and deteriorates the elastic fiber structures in the underlying dermis. (5) Topical application of THG ameliorates the structure of the basement membrane in ex vivo human skin explants*.* Taken together, THG might be a promising treatment for aged skin by suppressing the aberrant YAP-CCN1 axis.

## Introduction

Trehangelins (THG) are novel natural products derived from broth cultures of an endophytic actinomycete strain, *Polymorphospora rubra* K07-0510^1^. THG consist of a trehalose moiety and two angelic acid moieties and have a potent inhibitory activity against the hemolysis of red blood cells induced by light-activated pheophorbide a^1^. In a preliminary study, we evaluated the effect of THG on dermal fibroblasts using targeted DNA microarray analysis and found that THG down-regulated the mRNA expression level of CCN1, a ubiquitously expressed matricellular protein, and up-regulated COL1A1. This result suggested that THG modulate collagen homeostasis by regulating CCN1 expression^2^.

CCN1 is a member of the CCN family, deficiencies of which cause a failure in vasculogenesis during embryonic development^3^. Four structurally distinct domains characterize the function of CCN1: an insulin-like growth factor binding protein domain, a von Willebrand type C repeat domain, a thrombospondin type 1 repeat domain and a C-terminal cysteine-knot motif domain^4^. CCN1 promotes cell proliferation, survival and angiogenesis by binding to αvβ3-integrin, and it induces apoptosis and senescence through α6β1-integrin and proteoglycans^4^. In the dermis, levels of CCN1 increase with intrinsic aging and photoaging, and then drive senescence via the dysregulation of collagen metabolism^5–7^, which forms the age-associated dermal microenvironment^8^. In contrast, the level of CCN1 is elevated in the entire epidermis of the lesional skin of psoriasis patients and promotes the production of Interleukin-8 (IL-8), IL-1β and C–C Motif Chemokine Ligand 20, which drives psoriatic inflammation and skin barrier disruption^9–11^.

An epidemiological study showed that subjects complaining about “sensitive” or “very sensitive” skin were 2–4 times more likely to declare suffering from dermatosis^12^. Although often transient and in many cases unaccompanied by visible dermatological responses, sensitive skin can significantly affect the quality of life^13^. Generally, sensitive skin is thought to be hypersensitive to environmental factors and is vulnerable to skin problems^14^. The etiology of sensitive skin is still largely unknown, but sensitive skin is thought to be another skin condition related to the barrier function, together with neurosensory function, and other host and external factors. Saint-Martory et al. further identified a variety of external stimuli as triggers of sensitive skin, including ultraviolet (UV) exposure, wind, heat, cold, and pollution^15^.

The fact that CCN1 is associated both with epidermal inflammation and with dermal collagen homeostasis led us to hypothesize that CCN1 might play an important role in the barrier function and aging of sensitive skin. Moreover, the effect of THG on epidermal CCN1 in normal non-psoriatic skin remains unknown. Thus, the aim of this study was to clarify the characteristic roles of CCN1 in the epidermis and to identify an effective skin care solution that could be used to treat sensitive and aged skin.

The results of this study demonstrate that THG suppresses epidermal CCN1 by retaining Yes-Associated Protein (YAP) in the cytosol, which prevents its translocation to nuclei. Further, we demonstrate that excess secreted CCN1 in aged and stress-stimulated keratinocytes may induce an imbalance in epidermal growth and differentiation, inflammation and disruption of the extracellular matrix. We propose a possible mechanism for sensitive skin aging driven by CCN1 and a skin care solution by the topical application of THG.

## Results

### THG suppresses the expression of CCN1 in keratinocytes by inhibiting the translocation of YAP to the nucleus

**Figure 1:**
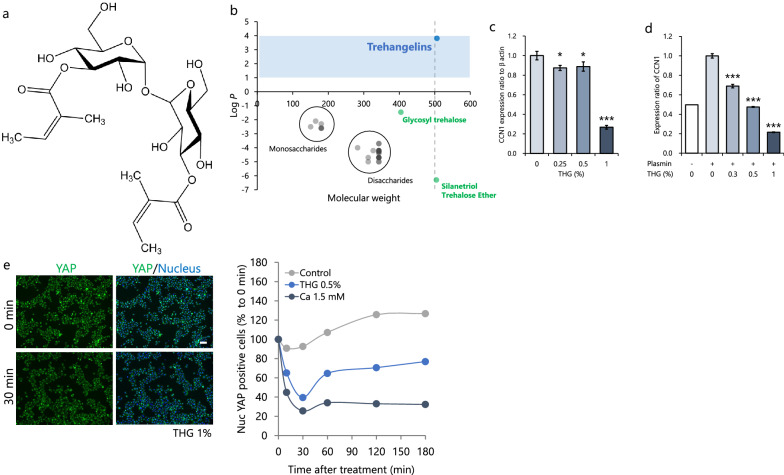
Trehangelins (THG) suppress the expression of CCN1 in keratinocytes by inhibiting the translocation of Yes-associated protein (YAP) to the nucleus. Structure of THG A (**a**). Estimated LogP of THG, Trehalose derivatives and mono/disaccharides was calculated using ChemDraw. Detailed list is shown in Supplementary Information (Table S1) (**b**). Effects of THG on CCN1 expression. Keratinocytes were pretreated with THG for 24 h and then were stimulated with or without 10 U plasmin, after which they were incubated for another 24 h. The expression of cellular CCN1 from the lysate (**c**) and secreted CCN1 from the supernatant (**d**) was determined by Western Blotting and was quantified. Values reported are means ± SD of n = 3 replicates, Dunnett’s test, *p < 0.05, **p < 0.01, ***p < 0.001 (**c**, **d**). Immunocytochemistry of YAP and analysis of nuclear YAP-positive cells 0–3 h after Ca or THG treatment (**e**). Values reported are means of n = 10,000 cells, Bar = 100 μm (**e**).

Three new trehalose compounds, designated THG A, B and C, were isolated from culture broth of an endophytic actinomycete strain, *Polymorphospora rubra* K07-0510. Although those compounds are structurally related, we used THG A in this study because of its lower cytotoxicity^1^. THG consist of a trehalose moiety and two angelic acid moieties (Fig. 1a). For compounds to penetrate into the skin, their deposition to the stratum corneum, the rate-limiting layer for skin penetration, is important. Generally, compounds with a LogP (octanol–water partition coefficient) of 1–4^16^ and with a molecular weight < 500 Da^17^ are thought to be suitable for permeability through the skin. Considering the physical properties of THG (MW: 506.5, LogP: 3.8), its penetration into the stratum corneum should be excellent (Fig. 1b). Thus, the effect of THG on keratinocytes was examined. Western blot analysis showed that treatment with THG significantly decreased the expression levels of cellular (Fig. 1c) and secreted CCN1 (Fig. 1d). It has been reported that YAP, a major downstream effector of the hippo signaling pathway, directly regulates the expression of CCN1^18^. Therefore, we next investigated whether THG suppress CCN1 via the YAP signaling pathway. Immunocytochemical analysis revealed that the translocation of YAP to nuclei was inhibited 30 min after treatment with THG, which might lead to the downregulation of CCN1 expression (Fig. 1e). These results indicate that THG is a potential suppressor of epidermal CCN1.

### Epidermal CCN1 regulates the balance of growth and differentiation of normal human keratinocytes

**Figure 2:**
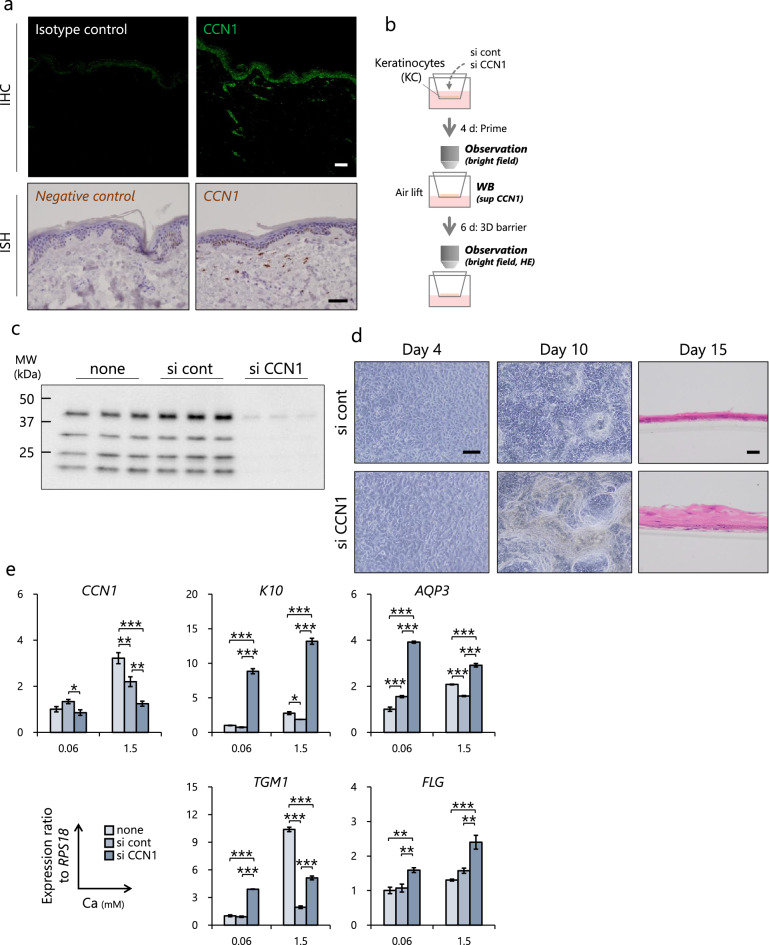

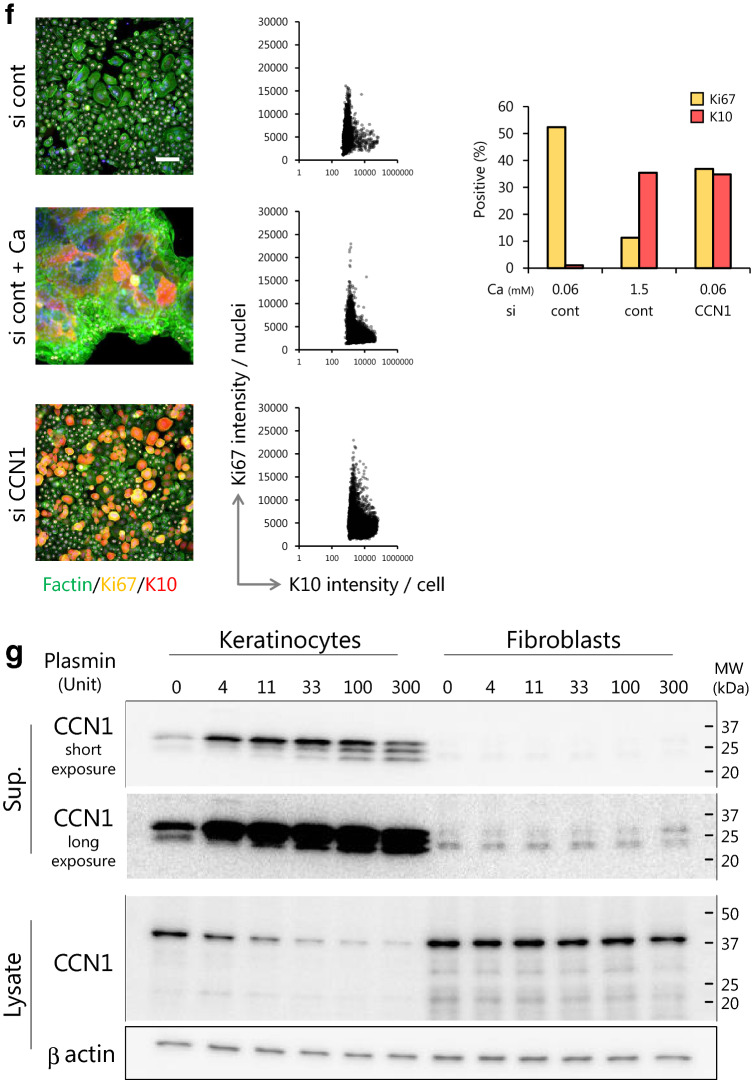
Epidermal CCN1 regulates the balance of growth and differentiation of normal human keratinocytes. CCN1 localization examined by immunohistochemistry (IHC) and by in situ hybridization (ISH) in human skin. Bars = 100 μm (**a**). Overview of the experimental method for the siRNA-transfected 3D epidermal model (**b**). Knockdown efficacy of CCN1 on day 4 determined by Western Blots of HPEKp supernatant samples. Full-length gels and blots are included in Supplemental Fig. 6a (**c**). Apical view of reconstructed skin before (day 4) and after (day 10) induction of differentiation, Bar = 300 μm (left). HE staining of reconstructed epidermis section, Bar = 20 μm (right) (**d**). Effects of CCN1 knockdown on mRNA levels of CCN1, Keratin 10, Aquaporin 3, Transglutaminase 1 and Filaggrin determined by qPCR. Total RNAs were collected from siRNA-transfected keratinocytes 6 h after Ca stimulation. Values reported are means ± SD of n = 3 replicates, Tukey’s test, *p < 0.05, **p < 0.01, ***p < 0.001 (**e**). Effects of CCN1 knockdown on Ki67 and K10 expression determined by imaging cytometry. Keratinocytes were fixed 48 h after transfection, Bar = 100 μm, n = 10,000 cells (**f**). Comparison of reactivity to plasmin treatment between keratinocytes and fibroblasts. Secreted (short and long exposure times) and cellular CCN1 levels were determined by Western Blots. Samples were collected 24 h after plasmin treatment. Full-length gels and blots are included in Supplemental Fig. 6b (**g**).

To determine whether CCN1 is involved in the epidermal homeostasis of normal non-psoriatic skin, we first examined its localization in the skin. Immunohistochemical analysis and in situ hybridization showed that CCN1 protein and mRNA are localized at the stratum basale in the epidermis as well as in the dermis (Fig. 2a). Thus, we next investigated the function of CCN1 using knockdown technology (Fig. 2b). Secreted CCN1 was clearly decreased in siCCN1-transfected keratinocytes (Fig. 2c) and slight morphological changes were observed in siCCN1-transfected keratinocytes (Fig. 2d, day 4). After inducing differentiation by changing the media to a high Ca concentration and air lifting, siCCN1-transfected keratinocytes were obviously cornified compared with untreated and siCont-transfected keratinocytes (Fig. 2d, days 10 and 15). qPCR analysis showed that the expression levels of 4 differentiation-related genes, K10, Aquaporin 3 (AQP3), Transglutaminase 1 (TGM1) and Filaggrin (FLG), were significantly upregulated in siCCN1-transfected keratinocytes (Fig. 2e). Moreover, imaging cytometry revealed that the expression of Ki67, a proliferation marker, was decreased while the expression of K10 was increased in siCCN1-transfected keratinocytes (Fig. 2f). As previously reported, CCN1 is a secreted matricellular protein that is cleaved by plasmin in lung epithelial cells^19^. Thus, the ability of keratinocytes to secrete CCN1 was compared with fibroblasts. Keratinocytes treated with plasmin showed decreased levels of full-length CCN1 (flCCN1: 42 kDa) and secreted more cleaved CCN1 (clCCN1: 25 kDa) than fibroblasts (Fig. 2g). These results indicate that clCCN1 secreted from keratinocytes might affect adjacent cells in autocrine and/or paracrine dependent manners.

### CCN1 secreted from keratinocytes induces inflammation and disrupts the mRNA expression of extracellular matrix constituents

**Figure 3:**
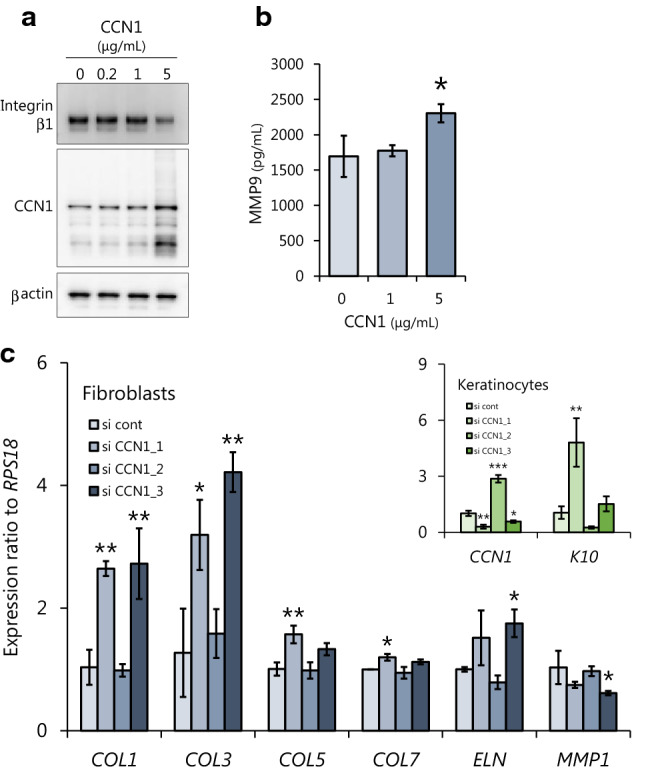
Secreted CCN1 from keratinocytes disrupts the expression of basement membrane and extracellular matrix components. Effects of excess CCN1 on inflammation of the stratum basale. Integrin-β1 and MMP9 expression in keratinocytes treated with human recombinant CCN1 for 48 h determined by western blotting and ELISA, respectively. Full-length gels and blots are included in Supplemental Fig. 6c (**a**, **b**). Effects of keratinocyte-derived CCN1 on mRNA expression levels of COL1, COL3, COL5, COL7, ELN and MMP1, in fibroblasts determined by qPCR. Three different sequences of siRNAs were used to avoid off-target effects (siCCN1_1-3). (**c**). Values reported are means ± SD of n = 3 replicates, Dunnett’s test, *p < 0.05, **p < 0.01, ***p < 0.001.

We next examined the inflammatory role of CCN1 focusing on molecules important for integrity of the basement membrane where CCN1 is highly expressed (as shown above). Keratinocytes treated with human recombinant CCN1 showed decreased expression levels of Integrin-β1 (Fig. 3a) and increased levels of MMP9 (Fig. 3b), which may induce disruption of the basement membrane. Next, the effect of secreted CCN1 on fibroblasts was evaluated using coculture assays. qPCR analysis revealed that the expression levels of extracellular matrix components, COL1, COL3, COL5, COL7 and elastin (ELN), were significantly upregulated in fibroblasts cocultured with siCCN1-transfected keratinocytes in a knockdown efficacy dependent manner (Fig. 3c). In contrast, MMP1 expression was significantly downregulated in those fibroblasts. From these results, we hypothesized that clCCN1 secreted from keratinocytes could make the dermal extracellular matrix structure more fragile.

### Epidermal CCN1 is increased by UVB exposure especially in aged keratinocytes and deteriorates the structure of elastic fibers in human skin

**Figure 4:**
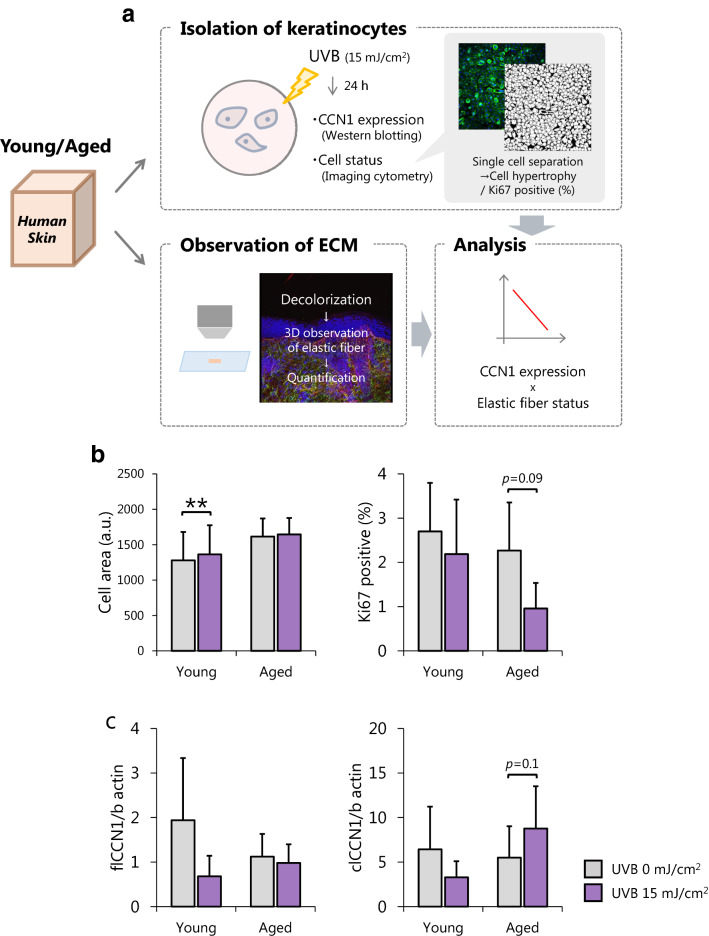

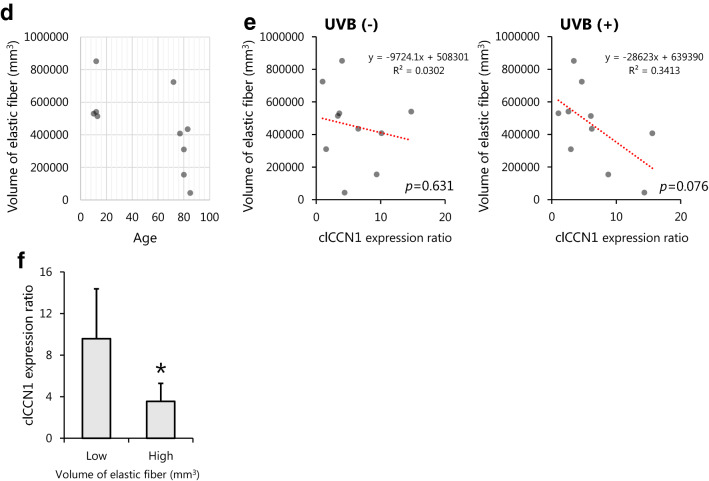
Epidermal CCN1 is increased by UVB exposure especially in aged keratinocytes and deteriorates the structure of elastic fibers in human skin. Overview of the experimental method (**a**). Cell area and Ki67-positive rate of isolated keratinocytes determined by imaging cytometry (**b**) and CCN1 expression (flCCN1: 42 kDa full length CCN1, clCCN1: 25 kDa secreted CCN1) in keratinocytes determined by Western Blots 24 h after 0 or 15 mJ/cm^2^ UVB irradiation. Values reported are means ± SD of n = 4 or 6, Student’s t test, **p < 0.01 (**c**). Volume of elastic fibers determined by immunohistochemistry of decolorized skin and structural analysis (**d**). Correlation analysis between clCCN1 expression and the volume of elastic fibers performed by Pearson's correlation test, n = 10 (**e**). Expression of clCCN1 in keratinocytes derived from volunteers with higher or lower elastin volumes. Values reported are means ± SD of n = 5, Student’s t test, *p < 0.05 (**f**).

To further test that hypothesis, we analyzed CCN1 expression in keratinocytes and the dermal structure derived from the same human skin (Fig. 4a). In this experiment, keratinocytes were exposed to UVB, which is one of the common triggers of sensitive skin. Imaging cytometry revealed that aged keratinocytes had an enlarged cell size and a decreased expression level of Ki67, indicating cellular senescence^20,21^, compared with young keratinocytes (Fig. 4b). The expression of CCN1, which is increased in aged fibroblasts, was unchanged between young and aged keratinocytes (Fig. 4c). However, after exposure to 15 mJ/cm^2^ UVB, a dose that induces oxidative stress, only aged keratinocytes showed upregulated levels of clCCN1 and evident proliferation arrest (Fig. 4b, c). To further confirm the effect of clCCN1 on the dermis, immunohistochemistry of the corresponding decolorized dermis was performed. The volume of elastic fibers was reduced in aged dermis (Fig. 4d). The expression of clCCN1 but not flCCN1 was negatively correlated with the volume of elastic fibers only in UVB-irradiated keratinocytes, although the difference was not statistically significant (*p* = 0.076) (Fig. 4e). We further compared the expression of CCN1 in keratinocytes derived from volunteers with higher or lower elastin volumes. Volunteers with lower volumes of elastic fibers had significantly higher expression levels of CCN1 (Fig. 4f). Overall, the secretion of CCN1 in the epidermis is likely to be caused by oxidative stress on aged keratinocytes, which leads to the deteriorated structures of elastic fibers.

### Treatment with THG ameliorates the structure of the basement membrane in ex vivo human skin explants

**Figure 5:**
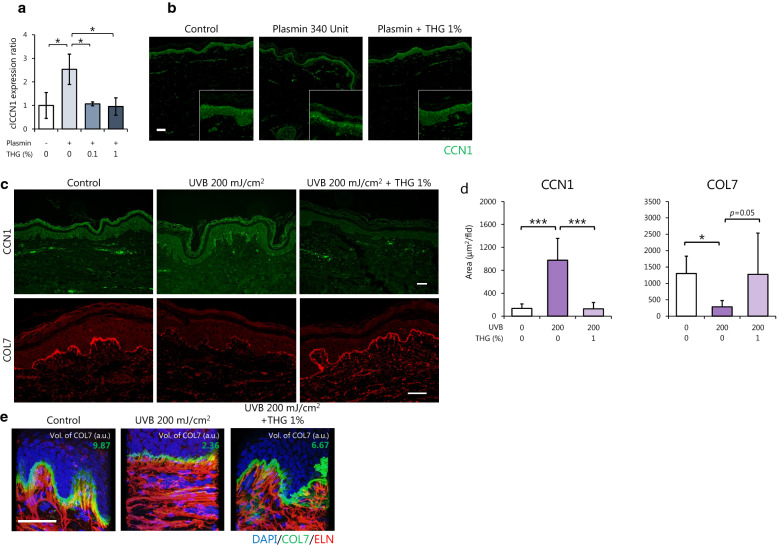
Treatment with THG ameliorates the structure of the basement membrane in ex vivo human skin explants. Effects of topically applied THG on CCN1 expression in plasmin-treated ex vivo skin explants. On day 1, 0–1% THG was applied on the stratum corneum and 340 U plasmin was applied to the medium. clCCN1 in the supernatant on day 5 was determined by Western Blots. Values reported are means ± SD of n = 3 replicates, Tukey’s test, *p < 0.05 (**a**). CCN1 expression and localization determined by immunohistochemistry, Bar = 100 μm (**b**). Effects of topically applied THG on the expression of CCN1 and COL7 in UVB irradiated ex vivo skin explants. 1% THG was topically treated from day 1. On days 1, 2 and 3, the inserts were exposed to 200 mJ/cm^2^ UVB. The expression of CCN1 and COL7 was determined by immunohistochemistry of skin sections collected on day 5, Bar = 50 μm (**c**). The area of CCN1 and COL7 around the basement membrane was quantified. Values reported are means ± SD of 9 fields from n = 3 replicates, Tukey’s test, *p < 0.05, ***p < 0.001 (**d**). The volume of COL7 and ELN was determined by immunohistochemistry of decolorized skin explants, Bar = 50 μm (**e**).

Finally, we confirmed the effects of THG on human skin explants. The treatment of skin explants with 340 U plasmin strongly enhanced the expression level of secreted CCN1, which was significantly decreased in topically THG treated skin explants (Fig. 5a, b). Since the downregulation of CCN1 enhanced the expression of extracellular matrix components including COL7 localized at the basement membrane (as shown above), the effect of THG on collagen was evaluated. UVB exposure of skin explants induced the expression of basal CCN1, which was significantly decreased in THG-treated explants (Fig. 5c, d). Furthermore, immunohistochemical analysis of decolorized skin revealed that the volume of COL7, which is disrupted by exposure to 200 mJ/cm^2^ UVB, was restored by the cutaneous application of THG (Fig. 5e). Taken together, the topical application of THG ameliorates the basement membrane via the downregulated secretion of CCN1 in the epidermis.

## Discussion

**Figure 6:**
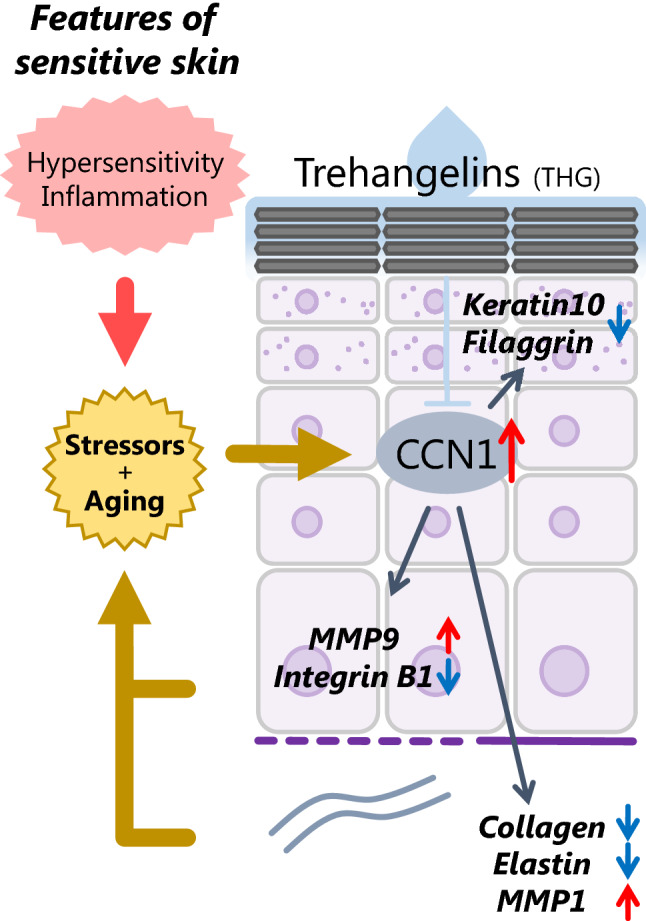
Graphical model of CCN1 driven aged and sensitive skin and the effect of THG.

In this study, we elucidated the function of epidermal CCN1, a factor that is important in psoriasis and in dermal senescence, and assessed the effects of THG on the skin (shown schematically in Fig. 6). Even in normal skin, CCN1 is localized at the stratum basale and is involved in the balance of growth and differentiation. Although YAP, a transcriptional coactivator that targets CCN1 expression, is known to maintain basal epidermal stemness^22,23^, this study demonstrated that the regulation of CCN1 alone can switch the balance of growth and differentiation. Furthermore, in the skin of patients with psoriasis, CCN1 is also involved in the hyperplasia of keratinocytes^24^. These facts imply that the function of the YAP-CCN1 axis in normal epidermis is likely to be consistent with psoriatic epidermis.

Treatment of keratinocytes with plasmin decreased levels of intracellular flCCN1 but markedly increased extracellular clCCN1 (Fig. 2f). Thus, we speculated that flCCN1 is digested intracellularly before its secretion as well as by the direct digestion of exosome-shuttled flCCN1 by extracellular plasmin^19^. Interestingly, keratinocytes secreted more clCCN1 than fibroblasts and the length of CCN1 fragments differed from each other (Fig. 2f). Because we performed western blotting to detect CCN1 using a monoclonal antibody to the N-terminus of CCN1, further studies are needed to identify the essential domain for skin senescence in the various CCN1 fragments derived from keratinocytes. These facts suggest that epidermis-derived CCN1 is indispensable for regulating the skin microenvironment and the distribution of flCCN1 and various CCN1 fragments may control the homeostasis of skin. Next, we found that the excess secretion of CCN1 from keratinocytes affects adjacent keratinocytes and fibroblasts. Because CCN1 itself is an agonist of integrin-β1^25^, excess CCN1 might desensitize integrin- β1. In combination with the upregulation of MMP9, the consequent disturbance of the basement membrane might enhance the penetration of CCN1 to the dermis. In spite of the general upregulation of collagen in fibroblasts cocultured with siCCN1-transfected keratinocytes, we couldn’t confirm the contribution of transforming growth factor-β signaling to that process (data not shown). Thus, it is conceivable that the disruption of extracellular matrix expression caused by epidermal clCCN1 might be mediated by the integrin-reactive oxygen species pathway^26^.

Furthermore, we successfully demonstrated the relationship between clCCN1 and the dermal structure by analyzing isolated keratinocytes and the corresponding dermis (Fig. 4). Using that strategy, our data clearly suggest that the epidermis, which is continuously exposed to environmental stresses, becomes vulnerable to those stresses with aging and affects tissues with a slow turnover like the dermis partly via epidermal CCN1. We would like to follow up our study to elucidate the behavior of CCN1 in sensitive skin in vivo and to clarify the relationship between clCCN1 and stimuli associated with sensitive skin other than UVB.

For a skin care solution, we discovered that THG is a suppressor of CCN1 in keratinocytes (Fig. 1). The transcription of CCN1 and the translocation of YAP are not affected by TXA (Supplemental data Fig. 1), which suggests that both TXA and THG suppress plasmin induced CCN1 by distinct mechanisms. Since trehalose doesn’t decrease CCN1 expression in keratinocytes and fibroblasts (data not shown), the two angelic acid moieties of THG must be important for the nuclear translocation of YAP. As the nuclear translocation of YAP was inhibited 30 min after THG treatment, it implies that THG might be directly involved in the upstream Hippo signaling pathway.

There are several possible limitations in this study. (1) The number of human skin samples was small and contributed to a lack of statistical power. (2) Unexpectedly, THG did not increase the expression of elastin in an ex vivo model. This would be influenced by the disadvantage of ex vivo cultures, which cannot be maintained for a sufficient length of time to show the histologic changes of elastic fibers. Although it is beyond the scope of this study, a previous study showed that the exogenous delivery of CCN1 or the induction of CCN1 signaling may have a therapeutic value for the treatment of fibrosis associated with wound healing^27^. Further dermatopharmacokinetic experiments are required to understand how to modulate the appropriate expression of CCN1 by THG.

In conclusion, THG is a promising candidate for suppressing the disrupted YAP-CCN1 signaling of epidermal keratinocytes. Since the concept of “sensitive skin” emerged in the 1970’s^28^, the population has steadily increased and has become an aging-conscious generation. We hope that these findings will provide a basis for the treatment of sensitive and senescent skin.

## Methods

### Reagents

Antibodies used in this study were as follows: anti-CCN1 (Cat# 14479S, Cell Signaling Technology, Danvers, MA, USA, for Western Blots, Cat# HPA029853, Sigma-Aldrich, St. Louis, MO, USA, for immunostaining), anti-Keratin10 (K10) (Cat# ab76318, Abcam, Cambridge, UK), anti-Ki67 (Cat# MU297-UC, Biogenex, Fremont, CA, USA), anti-integrin-β1 (Cat# MAB1965, Sigma-Aldrich), anti-Yes-Associated Protein (YAP) (Cat# sc-101199, Santa Cruz, Dallas, TX, USA), anti-type7 collagen (COL7) (Cat# ab93350, Abcam), anti-elastin (Cat# MAB2503, Abnova, Taipei, Taiwan), anti-β-actin (Cat# sc-47778, Santa Cruz), goat anti-rabbit IgG-HRP (Cat# A16104, Thermo Fisher Scientific, Waltham, MA, USA) and goat anti-mouse IgG-HRP (Cat# G-21040, Thermo Fisher Scientific). Alexa Fluor conjugated secondary antibodies were used for the visualization of immunostaining (Thermo Fisher Scientific). 4% Paraformaldehyde Phosphate Buffer Solution (4% PFA) (Cat# 161-20141) and plasmin solution from human plasma (Cat# 166-24231) were purchased from FUJIFILM Wako Pure Chemical Corp. (Osaka, Japan). The THG used in this study was a kind gift of NAGASE & CO., LTD (Tokyo, Japan).

### Human skin samples

Full-thickness normal human abdominal skin (from an 18 year old female) was obtained from CTI-biotech (Lyon, France) under ethical considerations. Samples were collected following informed consent of anonymous donor patients. Consenting and collecting procedures were compliant with European standards and applicable local ethical guidelines. Human eyelid skin (n = 4 young subjects: 10, 12, 12 and 13 years old, n = 6 aged subjects: 72, 77, 80, 80, 83 and 85 years old) was provided by the Kitasato University School of Medicine (Kanagawa, Japan). The study protocol conformed to the ethical guidelines of Kitasato University’s Ethics Committee (B16-285) and was conducted according to the principles of the Declaration of Helsinki. Informed consent was obtained from each patient or parent and/or legal guardian of minor subjects prior to enrollment in the study.

### Cell culture

Normal human epidermal keratinocytes (NHEKs, Thermo Fisher Scientific) were maintained in Gibco™ Epilife medium, with 60 μM calcium (Cat# MEPI500CA, Thermo Fisher Scientific) supplemented with Humedia-KG supplements (Cat# KK-6150, KURABO, Osaka, Japan). Primary human epidermal keratinocytes (HPEKp, CELLnTEC, Bern, Switzerland) were maintained in CnT-Prime Epithelial Culture Medium (Cat# CnT-PR, CELLnTEC) or in CnT-Prime 3D Barrier Culture Medium (Cat# CnT-PR-3D, CELLnTEC). For coculture assays, NHEKs were plated on transwell inserts and normal human dermal fibroblasts (NHDFs, Thermo Fisher Scientific) were plated on 12 well plates. Cells were maintained in EpilifeTM medium with supplements and 10% FBS containing Dulbecco’s Modified Eagle’s Medium (DMEM, Cat# 11995-065, Thermo Fisher Scientific), respectively. Eyelid skin-derived keratinocytes were obtained following treatment with Dispase I and 0.25% Trypsin/EDTA. The resulting cell suspension was diluted with DMEM containing 10% FBS, then was centrifuged and plated on COL1 coated dishes for more than 24 h. Keratinocytes obtained were then maintained as described above. Knockdown experiments were performed using Lipofectamine™ RNAiMAX Transfection Reagent (Cat# 13778075, Thermo Fisher Scientific) and Silencer Select^®^ Validated siRNAs (Cat#4390843: sicont, #s7244: siCCN1-1, #s7243: siCCN1-2, #s7242: siCCN1-3, Thermo Fisher Scientific) according to the manufacturer’s instructions. UVB irradiation was performed using a narrowband UVB lamp (Cat# TL 20W/01 RS SLV/25, PHILIPS, Amsterdam, Netherlands) in Hanks’ Balanced Salt Solution (+) after which the keratinocytes were incubated in fresh medium.

### Ex vivo skin explant culture

Excised skin was cultured according to the methods of Neil et al. and Portugal-Cohen et al. with slight modification^29,30^. Briefly, full-thickness normal human skin was processed within 24 h after excision. One cm square explants were placed on Netwell™ Inserts (Cat# 3477, Corning, NY, USA) and put in 12 well plates. DMEM:F12 1:1 (Cat# 11039-021, Thermo Fisher Scientific) with antibiotics added to each plate to keep the explants immersed but their surface dry (day 0). Twenty μL 1% THG solution in PBS was applied on the stratum corneum on day 1 and was replaced with fresh solution once a day. For plasmin stimulation, 340 U plasmin was systematically applied to the medium on day 1. For UVB irradiation, explants were transferred to 12 well plates with PBS(−) and were exposed to 200 mJ/cm^2^ UVB, after which they were put back into their original wells. After treatment with THG, plasmin or UVB, all samples were collected on day 5. All cells and explants used in this study were incubated in a humidified atmosphere with 5% CO_2_ at 37 °C. Validation of ex vivo cultures is shown in Supplemental Figs. 2–5.

### Reverse transcriptase-qPCR

Total RNAs were isolated from cells using a RNeasy^®^ mini kit (Cat# 74106, QIAGEN, Hilden, Germany), followed by reverse transcription to cDNAs using a PrimeScript^®^ RT reagent kit (Cat# RR037A, Takara Bio, Shiga, Japan) on a SympliAmp thermal cycler (Thermo Fisher Scientific). Real-time PCR reactions were performed using Applied Biosystems™ Power SYBR™ Green PCR Master Mix (Thermo Fisher Scientific) on a QuantStudio5^®^ (Thermo Fisher Scientific) with the respective primers (Perfect Real Time Primer, Takara Bio). Fold-changes of expression were calculated according to the ΔΔCT method using ribosomal protein S18 (RPS18) as an endogenous control.

### Western blotting

Whole cell extracts were prepared using 0.4% NP40 based cell lysis buffer and protein concentrations were determined using the BCA assay (Thermo Fisher Scientific). Whole cell extracts (1 μg/lane) were mixed with 4 × sample buffer and heated at 95 °C for 5 min. The samples were separated by 5–20% SDS-PAGE (Cat# NXV-376HP, DRC, Tokyo, Japan) and transferred to polyvinylidene difluoride membranes using a Trans-blot^®^ Turbo Transfer System (Biorad, Hercules, CA, USA). After incubation in StartingBlockTM (PBS) blocking buffer (Cat# 37578, Thermo Fisher Scientific) at room temperature for 15 min, the membranes were incubated with primary antibodies diluted in PBS containing 0.05% Tween20 (PBST) overnight at 4 °C. Proteins were visualized with corresponding secondary antibodies diluted with PBST (1:10,000) at room temperature for 1 h, followed by chemiluminescence detection using ECL Western Blotting Detection Reagents (#RPN2106, Cytiva, Tokyo, Japan) on LAS-4000 mini biomolecular imager (FUJIFILM Wako Pure Chemical Corp.).

### Immunocytochemistry and immunohistochemistry

Keratinocytes were fixed with 4% PFA at room temperature for 10 min. After blocking with StartingBlockTM (PBS) blocking buffer at room temperature for 30 min, keratinocytes were incubated with anti-K10 and anti-Ki67 antibodies diluted in Can Get Signal^®^ immunostain immunoreaction enhancer solution (Cat# NKB-501, TOYOBO, Osaka, Japan) overnight at 4 °C (1:1,000). Proteins were visualized with corresponding Alexa antibodies diluted with Can Get Signal^®^ immunostain immunoreaction enhancer solution (1:500), then counterstained with Alexa Fluor™ 488 Phalloidin (Cat# A12379, Thermo Fisher Scientific) and Hoechst 33342 at room temperature for 1 h, followed by observation using an IN Cell Analyzer 2200 (Cytiva) and analysis using the Developer toolbox (Cytiva). Skin tissues were fixed with 4% PFA, then embedded in paraffin and cut into 5 μm sections. Antigen retrieval was performed using Decloaking Chamber NxGen (BIOCARE Medical, Pacheco, CA, USA) at 95 °C for 10 min in pH 6.0 citrate buffer. After washing with PBS(-), slides were blocked and stained as described above using anti-CCN1 or anti-COL7 and corresponding Alexa antibodies. Samples were observed using FLUOVIEW FV1000 on an IX81 microscope (Olympus, Tokyo, Japan) or a BZ-X810 microscope (KEYENCE, Osaka, Japan) and was analyzed using a BZ-X800 analyzer (KEYENCE). For staining of decolorized skin, each fixed skin tissue was sectioned at a thickness of 1 mm in the direction of the epidermis-dermis, then washed with PBST and blocked with StartingBlock™ (PBS) blocking buffer. The sections were then incubated with anti-COL7 and anti-elastin antibodies diluted in StartingBlock™ (PBS) blocking buffer. After washing, sections were visualized with Alexa antibodies and DAPI diluted in PBS(−). After further washing, these sections were subsequently decolorized by the application of Rapiclear 1.49 (SunJin Lab Co., Hsinchu, Taiwan). Samples were observed using FLUOVIEW FV1000 on an IX81 microscope (Olympus, Tokyo, Japan). The volume of elastic fibers was calculated using Simpleware software (JSOL Corp., Tokyo, Japan) or Image J.

### Histomorphological observations

Reconstructed epidermal models and human skin specimens were fixed with 4% PFA, embedded in paraffin and cut into 5 μm sections. Histomorphological observations were performed after conventional Hematoxylin & Eosin (H&E) and Fontana-Masson staining.

### ELISA

The secretion of matrix metalloproteinase 9 (MMP9) into NHEK culture medium was determined using a Human MMP-9 DuoSet ELISA (Cat# DY911, R&D Systems, Minneapolis, MN, USA) and was performed according to the manufacturer’s instructions.

### In situ hybridization

CCN1 mRNA expression levels in tissues were detected using a RNAscope^®^ system (Advanced Cell Diagnostics, Newark, CA, USA) on 5 μm paraffin-embedded sections according to the manufacturer’s instructions.

### Nuclear translocation assay

Keratinocytes were immunostained as described above using anti-YAP and Alexa antibodies. After observation using a BZ-X810 microscope (KEYENCE), YAP-derived intensity localized in nuclei was analyzed using a BZ-X800 analyzer (KEYENCE).

### Statistical analysis

Statistical differences were assessed using EZR^31^ (Saitama Medical Center, Jichi Medical University, Saitama, Japan). Values reported represent means ± SD and *p* values of < 0.05 were considered statistically significant.

## Supplementary Information

Supplementary Table S1.

Supplementary Figures.

## Data Availability

The datasets generated during and/or analyzed during the current study are available from the corresponding author on reasonable request.
