# Supplementary material for: Trehangelins ameliorate inflammation-induced skin senescence by suppressing the epidermal YAP-CCN1 axis
Source: Sci Rep. 2022 Jan 19;12:952. doi: 10.1038/s41598-022-04924-6 (PMC8770704; doi:10.1038/s41598-022-04924-6)
Supplement: Supplementary file 2 — Supplementary Figures. [file 41598_2022_4924_MOESM2_ESM.pdf]

Trehangelins ameliorate inflammation-induced skin senescence by suppressing the epidermal YAP-CCN1 axis

Mami Yokota[1*], Yoshiyuki Kamiya[1], Tamie Suzuki[1], Shinsuke Ishikawa[2], Akira Takeda[2], Shinya Kondo[1], Takeshi Tohgasaki[1], Takuji Nakashima[3], Yoko Takahashi[4], Satoshi Ōmura[4] & Tetsuhito Sakurai[1]

[1]FANCL Research Institute, FANCL Corporation, 12-13 Kamishinano, Totsuka-ku, Yokohama, Kanagawa, Japan. [2]Department of Plastic and Aesthetic Surgery, Kitasato University School of Medicine, 1-15-1 Kitasato, Minami-ku, Sagamihara, Kanagawa, Japan. [3]Research Organization for Nano & Life Innovation, Waseda University, 530 Wasedatsurumaki-cho, Shinjuku-ku, Tokyo, Japan. [4]Ōmura Satoshi Memorial Institute, Kitasato University, 5-9-1 Shirokane, Minato-ku, Tokyo, Japan.

Supplemental figure 1

S1. a

S1. b

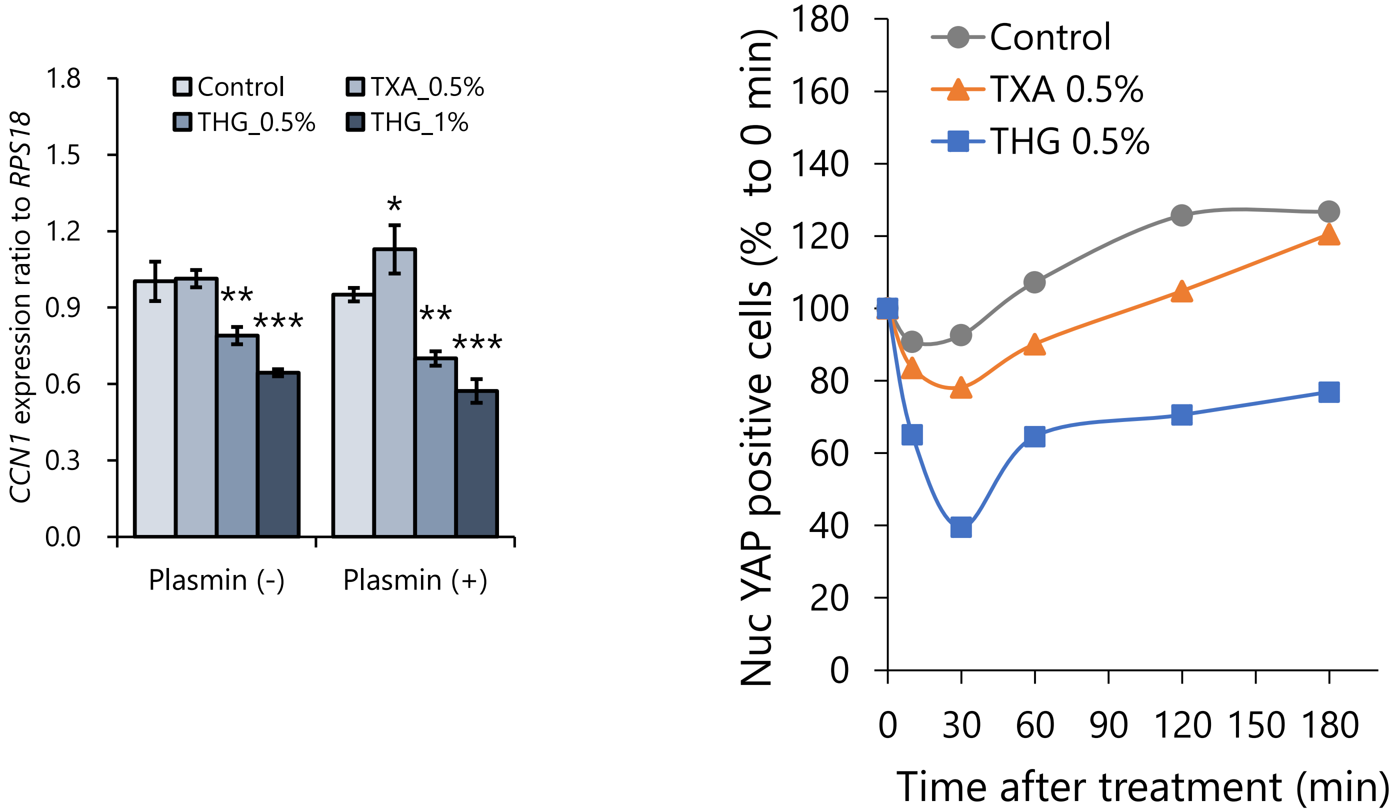

S1. Comparison of the mechanism of action between Tranexamic acid (TXA) and THG. RNA samples were collected after 3 h incubation with plasmin and/or THG, TXA, a competitive inhibitor of plasmin. CCN1 expression was determined by qPCR. Values reported are means ± SD of n=3 replicates, Dunnett's test, *p<0.05, **p<0.01, ***p<0.001 (a). Immunocytochemistry of YAP and analysis of nuclear YAP-positive cells 0-3 h after treatment with TXA or THG. Values reported are means of n=10,000 cells (b). These analyses revealed that THG, but not TXA, significantly decreased the mRNA expression level of CCN1 after 3 h of treatment.

Supplemental figure 2

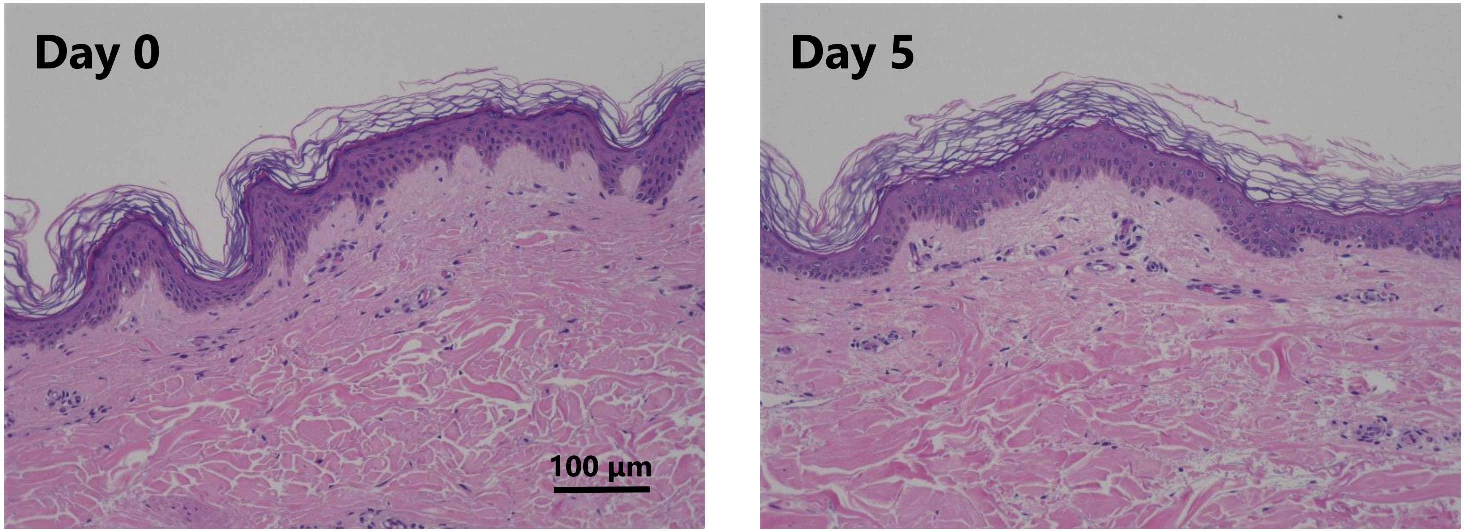

S2. Comparison of skin morphology between before and after *ex vivo* culture.
HE staining of skin samples immediately after excision (Day 0) and after 5 days of culture. Representative images of n=3 replicates; bar = 100 µm.

Supplemental figure 3

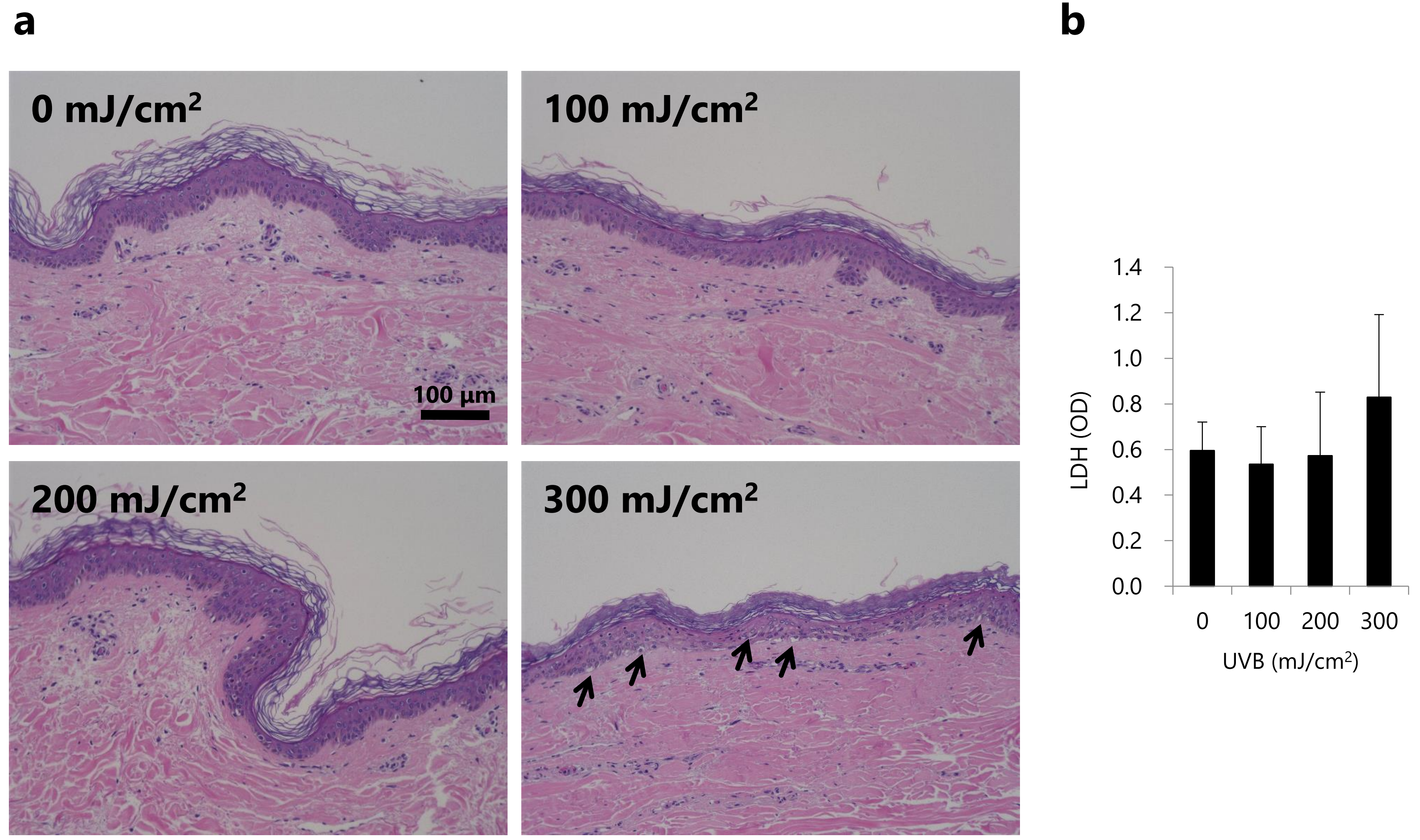

S3. The effect of UVB irradiation on skin morphology and cytotoxicity.
HE staining of UVB irradiated skin explants on day 5. The arrowheads point to areas of spongiosis. Representative images of n=3 replicates; bar = 100 µm (a). LDH activity of the culture media from each explants. Values reported are means ± SD of n=4 replicates, Dunnett's test (b).

Supplemental figure 4

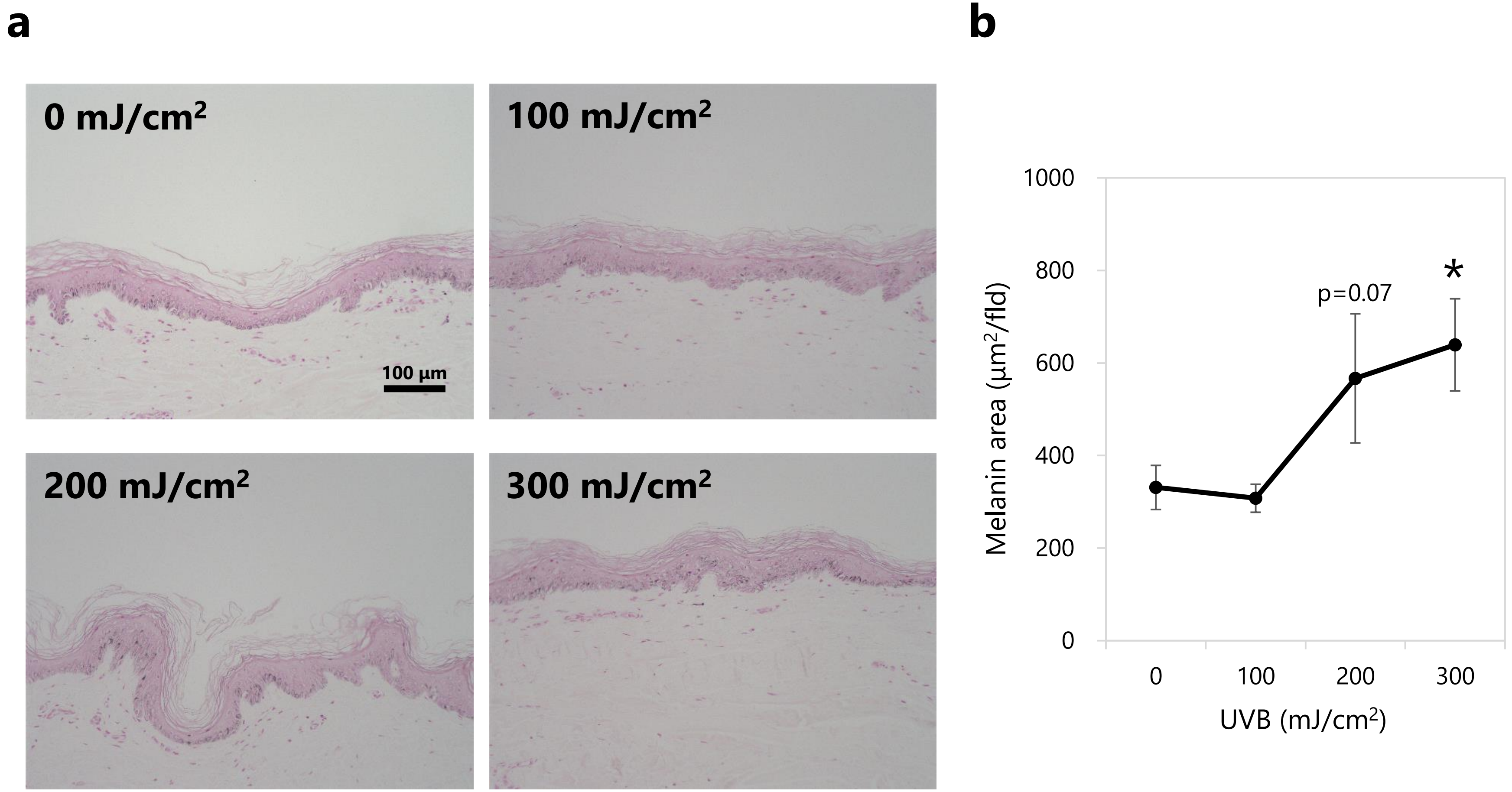

S4. The effect of UVB irradiation on melanogenesis.
Fontana-Masson staining of UVB irradiated skin explants on day 5. Representative images of n=3 replicates; bar = 100 µm (a). Quantification of melanin area. Values reported are means ± SD of n=3 replicates, Dunnett's test (b).

Supplemental figure 5

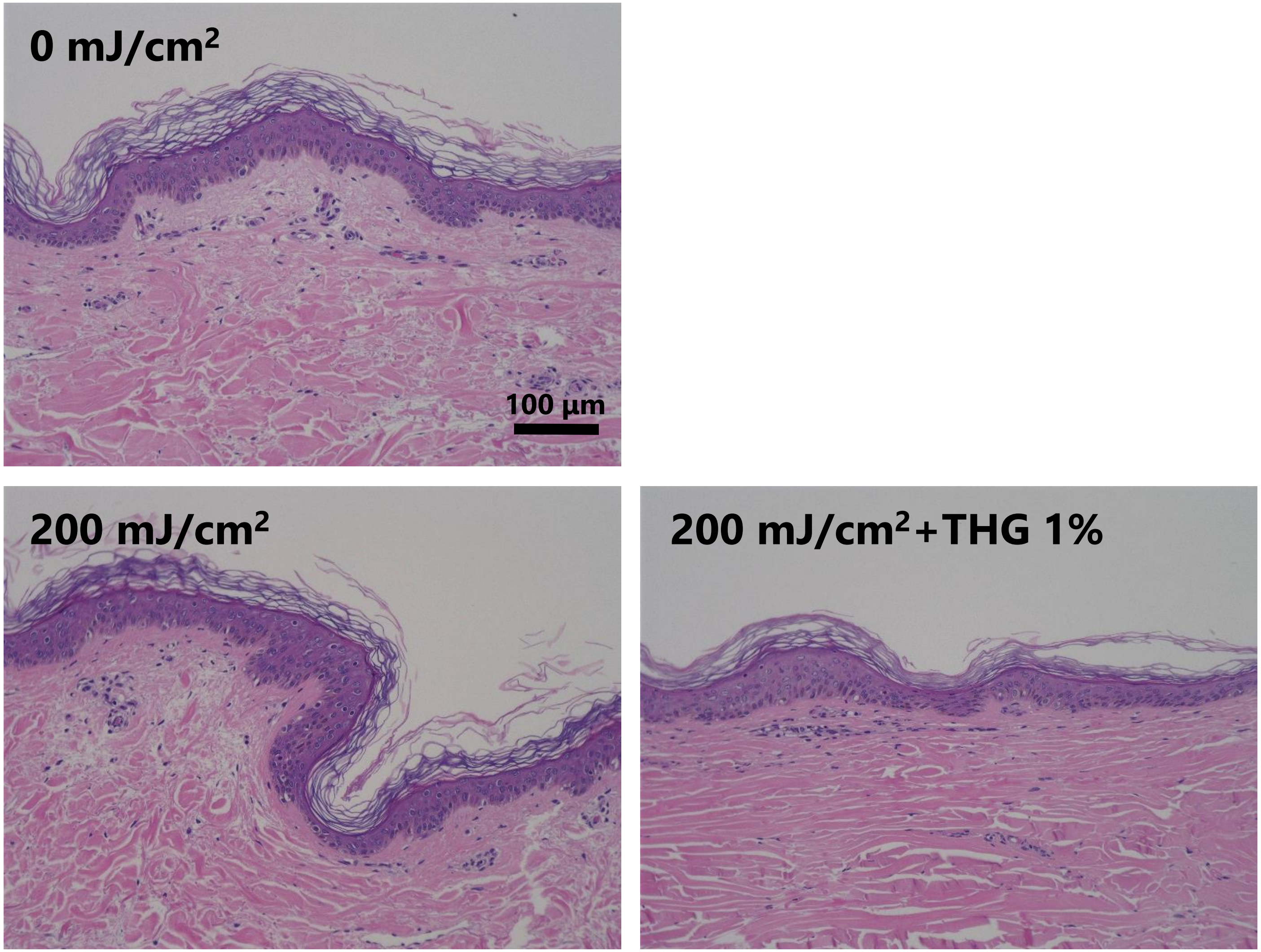

S5. The effect of THG on the morphology of UVB irradiated skin explants.
HE staining of UVB irradiated skin explants with/without THG treatment on day 5. Representative images of n=3 replicates; bar = 100 µm.

Supplemental figure 6

S6. a

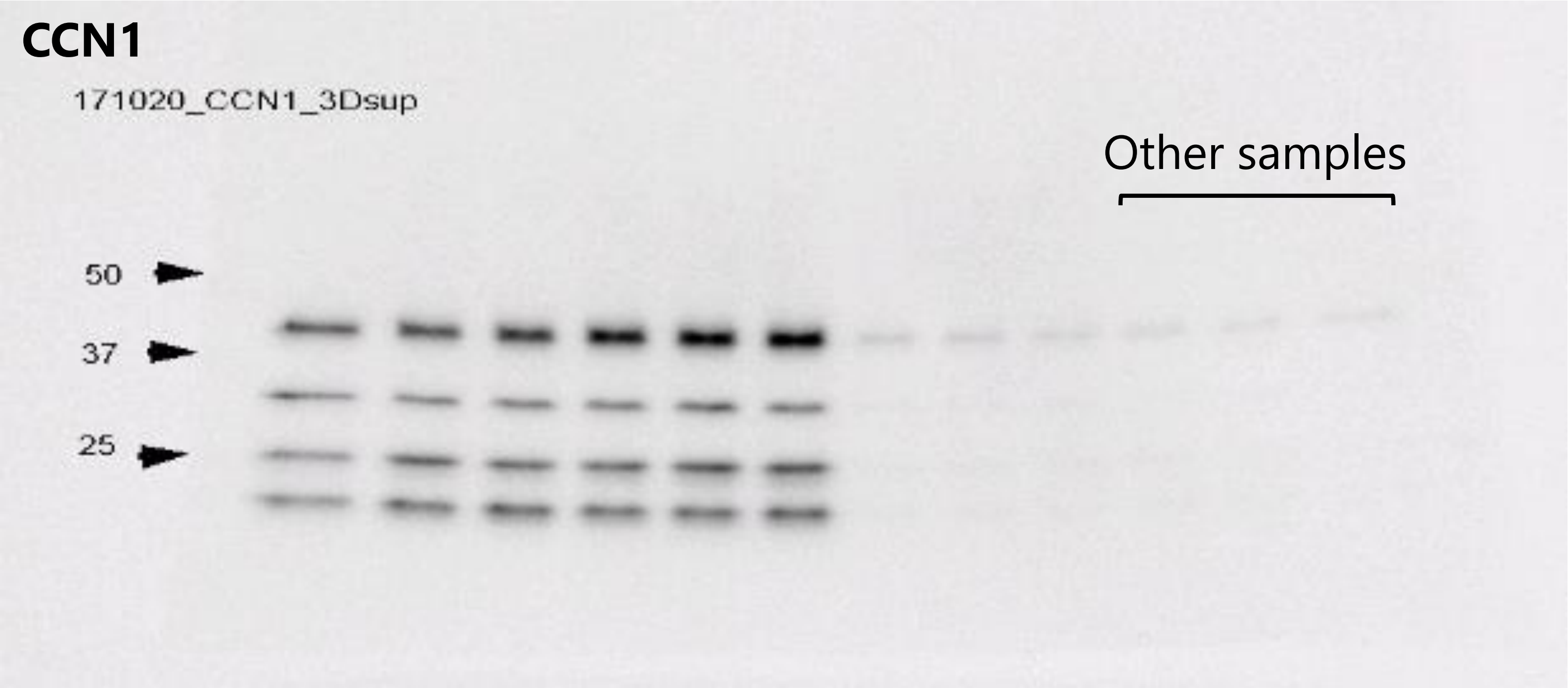

S6. Full-length blots shown in the main text. CCN1 blot from Fig. 2c (a), 2g (b), 3a (c).

Supplemental figure 6

S6. b

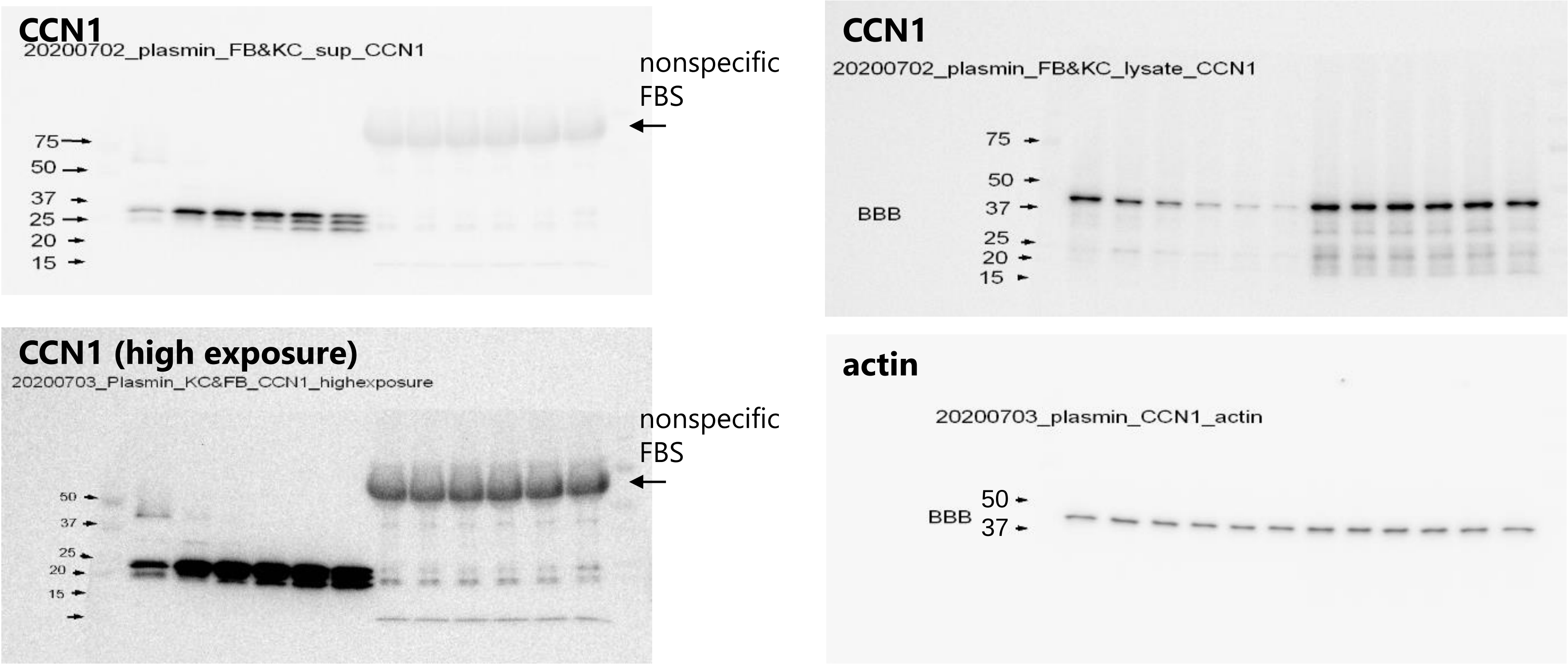

S6. Full-length blots shown in the main text. CCN1 blot from Fig. 2c (a), 2g (b), 3a (c).

Supplemental figure 6

S6. c

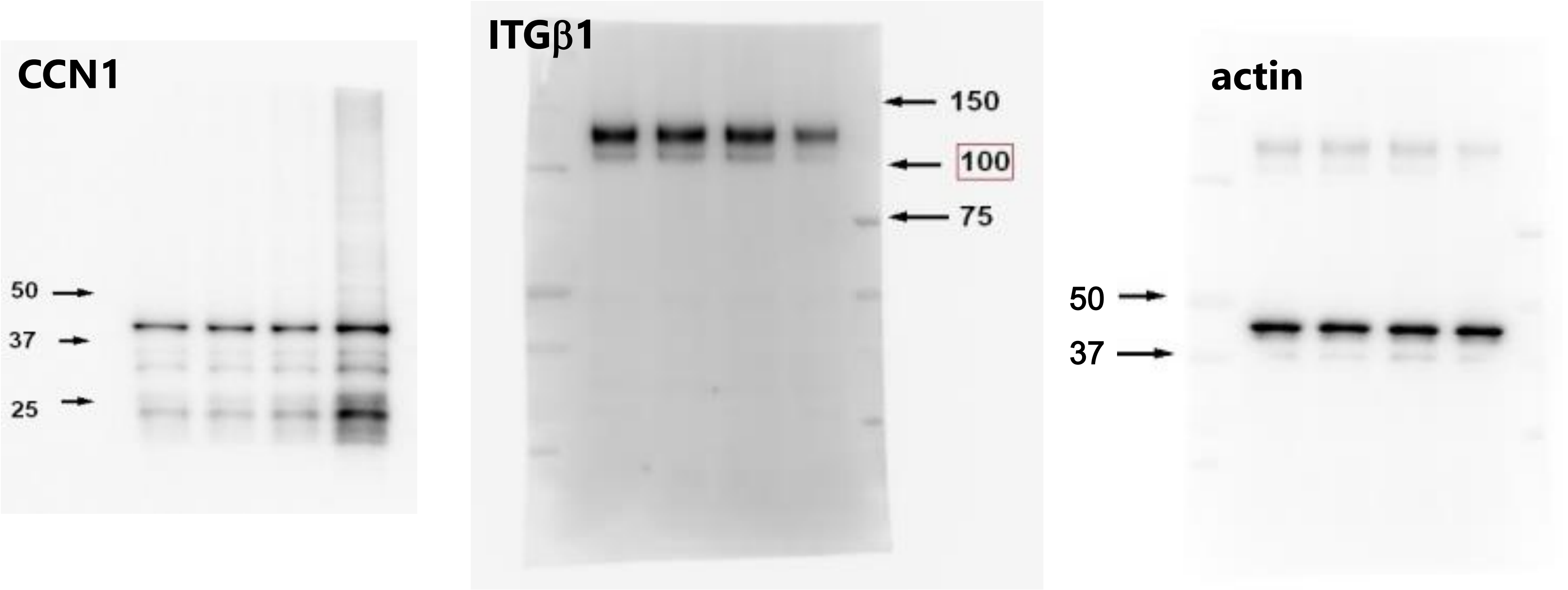

S6. Full-length blots shown in the main text. CCN1 blot from Fig. 2c (a), 2g (b), 3a (c).
